# MiR-128 Inhibits Tumor Growth and Angiogenesis by Targeting p70S6K1

**DOI:** 10.1371/journal.pone.0032709

**Published:** 2012-03-19

**Authors:** Zhu-mei Shi, Jing Wang, Zhiping Yan, Yong-ping You, Chong-yong Li, Xu Qian, Yu Yin, Peng Zhao, Ying-ying Wang, Xie-feng Wang, Ming-na Li, Ling-Zhi Liu, Ning Liu, Bing-Hua Jiang

**Affiliations:** 1 Department of Neurosurgery, The First Affiliated Hospital of Nanjing Medical University, Nanjing, China; 2 Department of Pathology, Cancer Center, Nanjing Medical University, Nanjing, China; 3 Department of Pathology, The First Affiliated Hospital of Nanjing Medical University, Nanjing, China; 4 Department of Pathology, Anatomy and Cell Biology, Kimmel Cancer Center, Thomas Jefferson University, Philadelphia, Pennsylvania, United States of America; H.Lee Moffitt Cancer Center & Research Institute, United States of America

## Abstract

MicroRNAs are a class of small noncoding RNAs that function as critical gene regulators through targeting mRNAs for translational repression or degradation. In this study, we showed that miR-128 expression levels were decreased in glioma, and identified p70S6K1 as a novel direct target of miR-128. Overexpression of miR-128 suppressed p70S6K1 and its downstream signaling molecules such as HIF-1 and VEGF expression, and attenuated cell proliferation, tumor growth and angiogenesis. Forced expression of p70S6K1 can partly rescue the inhibitory effect of miR-128 in the cells. Taken together, these findings will shed light to the role and mechanism of miR-128 in regulating glioma tumor angiogenesis via miR-128/p70S6K1 axis, and miR-128 may serve as a potential therapeutic target in glioma in the future.

## Introduction

MicroRNAs (miRNAs) are a class of endogenously expressed small noncoding RNAs that are usually 18∼23 nucleotides long, and regulate gene expression posttranscriptionally by targeting the untranslated regions [1]. MiRNAs have been shown to participate in a variety of cellular functions including cell apoptosis, cell proliferation, neural development, and stem cell differentiation [1]–[3]. They have been identified as a new kind of gene expression regulators through targeting mRNAs for translational repression or degradation [4]. Gliomas are the most common type of primary brain tumors in adults. Among gliomas, astrocytomas have the highest incidence [5]. According to World Health Organization's classification, astrocytomas are divided into four grades: pilocytic astrocytoma, diffuse astrocytoma, anaplastic astrocytoma, and glioblastoma multiforme (GBM). Anaplastic astrocytoma and glioblastoma multiforme are considered malignant glioma. Recently, many miRNAs are found to play important roles in glioma. MiR-7 was down-regulated in glioblastoma which inhibited invasiveness of primary glioblastoma lines [6], while miR-26 promotes glioblastoma cell proliferation *in vitro* and tumor growth *in vivo* by targeting several tumor suppressor genes such as PTEN and RB1 [7]. MiR-128 is a brain-enriched miRNA [8]. Most of the studies of miR-128 in carcinogenesis are focusing on glioma. For example, miR-128 has been shown to be down-regulated in glioma [8]–[10]. Overexpression of miR-128 inhibits cell proliferation by targeting E2F3a and Bmi-1, and reduces neuroblastoma cell motility and invasiveness through inhibiting Reelin and DCX [8], [10], [11]. However, further investigation needs to be performed to elucidate the role of miR-128 in carcinogenesis and tumor development of glioma.

P70S6K1 is one of the key downstream targets of mammalian target of rapamycin (mTOR), which plays important roles in cancerous characteristics such as cell cycle, cell apoptosis, cell growth and proliferation [12], [13]. Growing evidence indicates that p70S6K1 pathway is involved in carcinogenesis, metastasis and chemotherapeutic drug resistance [12]–[14]. Our previous studies have demonstrated that mTOR/p70S6K1 is involved in regulating tumor angiogenesis and tumorigenesis [15]–[17]. Tumor angiogenesis is required for tumor development and growth. Vascular endothelial growth factor (VEGF) is considered to be the most important growth factor among the angiogenic factors [18], [19]. Hypoxia-inducible factor 1 (HIF-1) is one of the major regulator of VEGF [20]. High levels of HIF-1 expression are observed in many human cancers, and correlated with tumorigenesis [21]. P70S6K1 is implicated in regulating HIF-1α expression [15], [16], [22], [23]. However, it is unclear what miRNAs directly regulate p70S6K1 in glioma.

In the present study, we found that miR-128 was down-regulated in human glioma and acted as a tumor suppressor by directly targeting p70S6K1. Forced expression of miR-128 inhibited p70S6K1, HIF-1α, and VEGF expression. Overexpression of p70S6K1 restored miR-128-inhibited HIF-1α and VEGF expression, confirming that p70S6K1 is the downstream target of miR-128. In addition, miR-128 overexpression suppressed cell proliferation and attenuated tumor growth and angiogenesis *in vivo*. These results give novel insights into our understanding the role and mechanism of miR-128 in glioma pathoetiology, and provide a potential therapeutic strategy for glioma treatment in the future.

## Materials and Methods

### Human tissue samples

All human normal brain and glioma tissue samples were obtained from the Department of Neurosurgery, the first affiliated hospital of Nanjing Medical University. This study procedure was approved by The Institutional Review Board at the hospital. All participants provided written informed consent. Tissue samples were collected during surgery. For each sample, the major portion of tissue was frozen immediately in liquid nitrogen for molecular analysis, and the remaining tissue was fixed in paraformaldehyde for histological examination. All samples were histologically classified and graded according to WHO guidelines by clinical pathologist, and were prepared for cases in the institute biorepository, and classified and selected based on diagnosis. No information regulated by The Health Insurance Portability and Accountability Act was included in the study, which qualifies for the status of USA NIH Exemption # 4.

### Cell culture and reagents

Human glioma cell lines, U87 and U251 were cultured in DMEM medium supplemented with 10% fetal bovine serum (FBS), 100 units of penicillin/ml, and 100 ng of streptomycin/ml. HEK-293 cells were cultured in DMEM medium supplemented with 10% FBS, 100 units of penicillin/ml, 100 ng of streptomycin/ml., and 2 mmol/ml glutamine. All cells were incubated at 37°C supplemented with 5% CO_2_. Antibodies against HIF-1α and HIF-1β, and the growth factor-reduced Matrigel were from BD Biosciences (Bedford, MA, USA). The antibodies against phospho-p70S6K1 (Thr389), p70S6K1 were from Cell Signaling Technology (Beverly, MA, USA). Monoclonal antibody against β-actin was from Sigma. Antibodies against GAPDH were from Kangcheng Company (Shanghai, China).

### Lentiviruses packaging and stable cell lines

The hsa-miR-128 and hsa-miR-scrambled control (miR-SCR) lentiviral constructs were purchased from Thermo Fisher Scientific (Huntsville, AL, USA). To establish stable cells expressing miR-128 and miR-SCR, lentiviruses were packaged using Lentiviral Packaging System according to the manufacturer's instructions (Thermo Fisher Scientific). The lentiviral supernatant obtained from HEK-293 cells was used to transduce U87 and U251 cells. Stable cell lines were selected by puromycin.

### RNA extraction, reverse transcription PCR and quantitative real time- PCR

Total RNAs were extracted from cells with Trizol (Invitrogen, CA, USA). One microgram of RNAs was used for cDNA synthesis using oligo(dT)_15_ and M-MLV reverse transcriptase. Aliquots of cDNAs were amplified with primers for VEGF and GAPDH. The primers are as follows: VEGF forward primer, 5′-TCGGGCCTCCGAAACCATGA-3′; VEGF reverse primer, 5′-CCTGGTGAGAGATCTGGTTC-3′; GAPDH forward primer, 5′-CCACCCATGGCAAATTCCATGGCA-3′; GAPDH reverse primer, 5′-TCTAGACGGCAGGTCAGGTCCACC-3′. VEGF and GAPDH were amplified by PCR for 30 cycles with each cycle at 95°C for 1 min, 59°C for 30 s, and 72°C for 1 min. The relative VEGF mRNA levels were normalized to those of GAPDH mRNA levels using Quality One analysis software (Bio-Rad, USA).

To analyze miR-128 expression levels, RNAs were extracted from tissues. The stem-loop RT-PCR assay was used to quantify the miRNAs expression levels as described previously [24], [25]. The RT-PCR primers were as following: miR-128 RT primer: 5′-CTCAACTGGTGTCGTGGAGTCGGCAATTCAGTTGAGAAAAGAGA-3′. miR-128 PCR primers: sense: 5′-ACACTCCAGCTGGGTCACAGTGAACCGGTC-3′; anti-sense: 5′- TGGTGTCGTGGAGTCG-3′. U6 RT primer: 5′-TGGTGTCGTGGAGTCG. U6 PCR primers: Sense: 5′-CTCGCTTCGGCAGCACA; anti-sense: 5′-AACGCTTCACGAATTTGCGT. PCR products were separated on 2.0% agarose gels stained with ethidium bromide, and visualized under UV light. RNA input was normalized to the level of human U6 snRNAs. qRT-PCR was performed using SYBR Premix DimerEraser (Takara, Dalian, China) on a 7900HT system. MiR-128 and U6 PCR primers were the same as described previously. The expression levels of miR-128 were normalized with reference to expression levels of U6 snRNA, and fold changes were calculated by relative quantification (2^−ΔΔCt^) [26].

### Vector construction

pRK7-HA-S6K1 expression vector was as previously described [27]. The p70S6K1 3′-UTR sequence was amplified from human cDNAs by PCR using the following primers: p70S6K1 forward primer, 5′-CGCGGATCCGTAAATGGCTTGTGATACTCTTGA-3′; p70S6K1 reverse primer, 5′-CGCCTCGAGATAGGTGTGTGCTTTCTGTCTGTG-3′. For its mutagenesis, the sequence complementary to the binding site of miR-128 in its 3′UTR (CACTGTGA) was replaced by CAGTCTCA. The wild-type and mutated 3′-UTRs of p70S6K1 were cloned into pMIR-REPORT miRNA reporter vector using the Sacl and HindIII sites. These constructs were validated by sequencing.

### Luciferase assay

HEK-293 cells were seeded into a 24-well plate. After cultured overnight, cells were co-transfected with the wild-type and mutated p70S6K1 3′-UTR reporter plasmid, and pRL-TK plasmids, or transfected with pre-miR-128 and miR-scrambled control precursors (miR-SCR). Luciferase assays were performed 48 h after transfection using the Dual Luciferase Reporter Assay System (Promega, WI, USA).

### MiRNA precursor transfection

Pre-miR-128 and miR-SCR precursors were purchased from Applied Biosystems (Carlsbad, CA). Cells at 50–70% confluence were transfected using lipofectamine reagent (Invitrogen, CA, USA). Transfection complexes were prepared according to the manufacturer's instructions. The final concentration of miR-128 or miR-SCR precursor for the transfection was 40 nmol/L.

### Immunoblotting

After specific treatments as indicated, cells were washed with ice-cold PBS buffer, scraped from the dishes, and centrifuged. The supernatant was collected as the total cellular protein extracts and stored at −80°C. Cell lysates were prepared using RIPA buffer supplemented with protease inhibitors (100 mM Tris, pH 7.4, 150 mM NaCl, 5 mM EDTA, 1% Triton X-100, 1% deoxycholate acid, 0.1% SDS, 2 mM phenylmethylsulfonyl fluoride, 1 mM sodium orthovanadate, 2 mM DTT, 2 mM leupeptin, 2 mM pepstatin). Tumor tissues from human and nude mice were grinded into powder in liquid nitrogen with RIPA buffer, and the total tissue proteins were extracted as described above. Aliquots of protein lysates were fractionated by SDS-PAGE, transferred to a nitrocellulose membrane (Whatman, Germany), and subjected to immunoblotting analysis according to the manufacturer's instruction. ECL Detection System (Thermo Scientific) was used for signal detection.

### ELISA assay for VEGF

The protein levels of VEGF in the supernatant were measured using the Quantikine human VEGF ELISA kit (NeoBioscience, Shanghai, China) according to the manufacturer's instruction. In brief, the cells were seeded in 6-well plates and cultured to 90% confluence, and then cells were switched to fresh medium. The supernatants were collected and the number of cells in each well was counted after 24 h. VEGF in the supernatant (100 µl) was determined, and normalized to the cell number. A serial dilution of human recombinant VEGF was included in each assay to obtain a standard curve.

### Cell proliferation assay

To determine the effects of miR-128 on the cell proliferation, U87 and U251 cells (6×10^4^ cells) were seeded in a 6-well plate and cultured overnight. The cells were transiently transfected with pre-miR-128 and miR-SCR precursors, respectively; and cultured overnight. Then the cells were trypsinized and seeded at 2000 cells per well in a 96-well plate. The proliferation of the cells was measured using a Cell Counting Kit-8 (CCK-8) (Dojindo Laboratories, Kumamoto, Japan) according to the manufacturer's instruction every 24 h after transfection. Three independent experiments were done in triplicate.

### Tumorigenesis in nude mice

Male BALB/cA-nu nude mice (4-weeks-old) were purchased from Shanghai Experimental Animal Center (Chinese Academy of Sciences, Shanghai, China), and maintained in pathogen-free conditions. Eight mice were randomly divided into two groups. U87 cells stably expressing miR-128 were injected subcutaneously into both flanks of nude mice (5×10^6^ cells in 100 µl). U87 cells stably expressing miR-SCR were used as the negative control. Bi-dimensional tumor measurements were measured with calipers three times weekly. Tumor volumes were calculated according to the formula (width^2^×length)/2. The mice were euthanized after 28 days, and tumors were weighed.

### Matrigel plug assay

Male BALB/cA-nu nude mice (6-weeks-old) were purchased from Shanghai Experimental Animal Center (Chinese Academy of Sciences, Shanghai, China), and maintained in pathogen-free conditions. Eight mice were randomly divided into two groups. U87 cells stably expressing miR-128 were harvested and resuspended in serum-free medium. Aliquots of the cells (2×10^6^ cells in 100 µl) were mixed with 200 µl of Matrigel. The mixture was immediately injected into both flanks of nude mice. U87 cells stably expressing miR-SCR in equal volumes of solvent were used as the control. On Day 11 after the implantation, the Matrigel plugs were trimmed out and used for the measurement of the hemoglobin content using Drabkin's Reagent Kit (Sigma-Aldrich, St Louis, MO) according to the manufacturer's instructions. The concentrations of hemoglobin were calculated based on a set of hemoglobin standards.

### Immunohistochemical analysis

Tumors samples were fixed in Bouin's solution and embedded in paraffin. Tumor sections at 5 µm were cut and deparaffinized, and antigen was retrieved by the heating using microwave for 15 min. After incubation with hydrogen peroxide, the sections were washed with 1×PBS buffer for three times. The sections were blocked for 1 h with 5% Albumin Bovine serum in PBS buffer. Antibodies against HIF-1α (Bioworld, China), VEGF(Santa Cruz, CA, USA) and anti-Human CD31 (Abcam, USA) were used in a humid chamber at 4°C for 16 h. After washing three times, the sections were incubated with EnVision+, Peroxidase, Rabbit (Gene Tech, Shanghai, China) for 2 h. The protein signals were detected using 3,3′-diaminobenzidine(DAB) (Gene Tech, Shanghai, China). Different sections were prepared from three of the Matrigel plugs in every group, and the microvessels were counted in three different fields per section as follows: slides were first scanned under low power (×100) to determine three “hotspots” or areas with the maximum number of microvessels, then the positive-stained blood vessels in the selected areas were analyzed at ×400 magnification [17].

### Statistical analysis

All values in this study were presented as mean
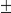
SD. Statistical analysis was performed based on a Student's t-test at the significance level of P<0.05. Spearman's correlation analysis was used to determine the correlation of the expression levels of p70S6K1, miR-128, and the CD31-positive microvessel densities using SPSS software.

## Results

### MiR-128 expression was decreased in gliomas

**Figure 1:**
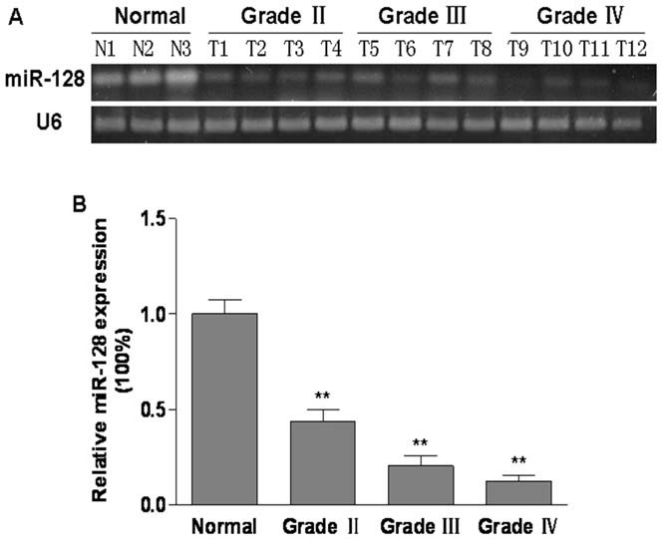
MiR-128 was down-regulated in human glioma tissues when compared to normal brain tissues. (A) Semi-quantative RT-PCR analysis of miR-128 levels in three normal brain tissues (N1–N3) and twelve glioma tissues (T1–T4 Grade II, T5–T8 Grade III, T9–T12 Grade IV). U6 RNA level was used as an internal control. (B) Relative miR-128 expression levels were analyzed by qRT-PCR in normal brain tissues and three grades of glioma tissues (Grade II, Grade III, Grade IV). U6 RNA level was used as an internal control. Double asterisks indicate significant difference when compared to normal brain tissues (P<0.01).

To determine the levels of miR-128 in glioma and normal brain tissues, total RNAs were extracted from normal brain tissues and glioma tissues at Grades II, III, and IV, and the expression levels of miR-128 were analyzed using RT-PCR and real time PCR. As shown in Figure 1A, the expression levels of miR-128 were down-regulated in glioma tissues when compared to the normal brain tissues. The levels of miR-128 expression were quantified using eight normal brain tissues, glioma tissues at Grades II, III, and IV with thirteen cases each, and normalized to the U6 levels. The levels of miR-128 expression in glioma tissues were significantly decreased when compared with normal tissues (Figure 1B). There was no significant difference of miR-128 expression levels in glioma tissues between Grade III and Grade IV, which was significantly lower than that in Grade II (Figure 1B). This result indicates that miR-128 is down-regulated in glioma.

### P70S6K1 was a direct target of miR-128

**Figure 2:**
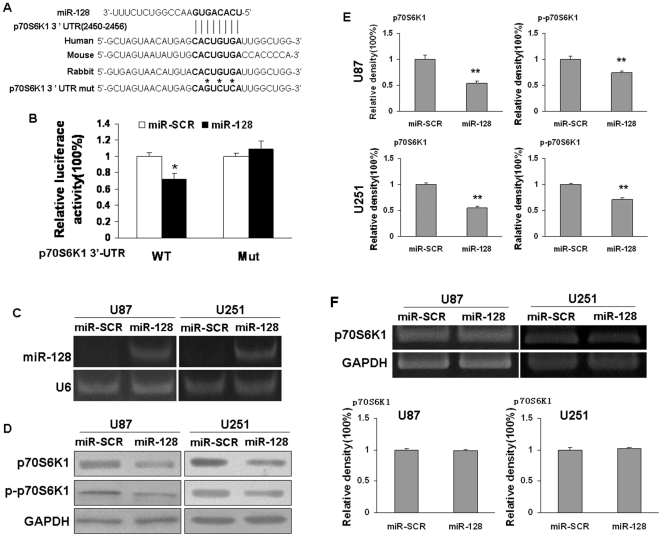
MiR-128 directly targeted p70S6K1. (A) Schematic diagram of putative miR-128 binding site in the 3′-UTR regions of p70S6K1 in human, mouse and rabbit. The seed sequence of miR-128 matches 3′-UTR regions of p70S6K1 (in bold). The mutated nucleotides of the p70S6K1 3′-UTR was labeled by asterisk. (B) Relative luciferase activities of p70S6K1 wild type (WT) and mutant (Mut) 3′-prime UTR regions were obtained by co-transfection of scrambled control miRNA or miR-128 precursor, and pRL-TK plasmid; and calculated as the ratio of firefly/renilla activities in the cells and normalized to those of the control. The results were presented as mean±SD from three independent experiments with each experiment in triplicate. Asterisk indicates significant difference when compared to control (P<0.05). (C) U87 and U251 cells stably expressing scrambled control (SCR) and miR-128 were subjected to RT-PCR analysis for miR-128 and U6 expression levels. (D) Overexpression of miR-128 inhibited p70S6K1 expression at protein level. The expression levels of total p70S6K1, phospho-p70S6K1 (Ser389), and GAPDH protein in U87 and U251 cells stably expressing miR-SCR and miR-128 were analyzed by immunoblotting. (E) Relative signals were quantified using VisionworksLS Acquisition and analysis software. Results are presented as mean±SD from three independent experiments. Double asterisks indicate significant difference when compared to the control (P<0.01). (F) miR-128 overexpression did not change the p70S6K1 mRNA level detected by RT-PCR. Relative densities were quantified using VisionworksLS Acquisition and analysis software.

To better understand molecular action of miR-128 in glioma, we searched for potential targets of miR-128 by TargetScan, and tested the potential targets using bioinformatic database. We found that miR-128 has a putative binding site to the 3′-UTR region of p70S6K1 with 100% conserved sequence in human, mouse, and rabbit (Figure 2A). The p70S6K1 3′-UTR was cloned into pMIR-REPORT miRNA reporter vector. Overexpression of miR-128 decreased luciferase activity of this reporter to 70% of the control level (Figure 2B), suggesting that miR-128 inhibits the 3′-UTR function of p70S6K1. To test whether miR-128 specifically inhibited p70S6K1 by its potential binding site of seed sequence, the mutated reporter at miR-128 binding site was constructed. Forced expression of miR-128 did not affect the mutant p70S6K1 reporter activities (Figure 2B). These results indicate that p70S6K1 is a direct target of miR-128 with the specific binding site at the seed sequence.

Next, we established cell lines stably expressing miR-128 and its scrambled control (miR-SCR) using human glioma cells U87 and U251 by lentiviral transduction. The expression levels of miR-128 were analyzed in these stable cell lines using RT-PCR. As shown in Figure 2C, the expression levels of miR-128 were increased in pLe-miR-128-expressing U87 and U251 cells, respectively, demonstrating that these stable cells successfully expressed miR-128. The cells with miR-128 overexpression showed low levels of p70S6K1 and p-p70S6K1 proteins by 30–50% reduction when compared to those of scrambled control cells (Figures 2D and 2E). In addition, forced expression of miR-128 did not change p70S6K1 mRNA expression levels (Figure 2F), showing that miR-128 specifically regulates p70S6K1 protein expression at posttranscriptional levels.

### P70S6K1 levels inversely related to the levels of miR-128 expression in glioma tissues

**Figure 3:**
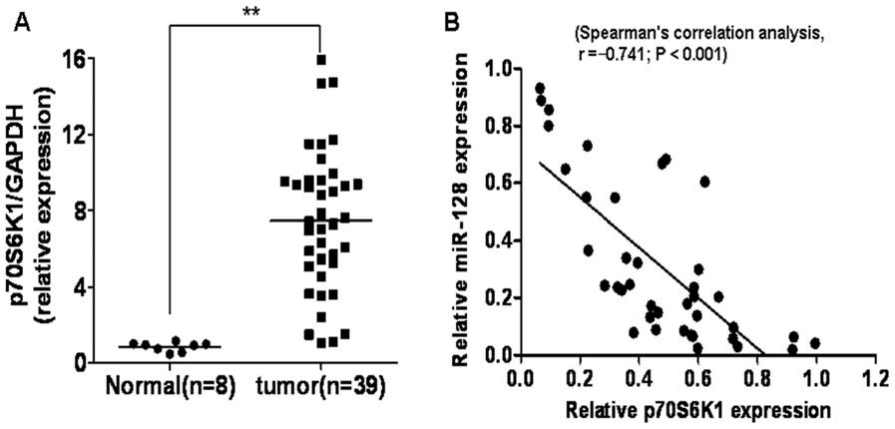
The p70S6K1 protein levels were negatively correlated with miR-128 levels in glioma tissues. (A) The expressions of p70S6K1 in human glioma specimens and normal brain tissues were determined by immunoblotting and the fold changes were obtained by the ratio of p70S6K1 to GAPDH level. Double asterisks indicate significant difference when compared to normal brain tissues (P<0.01). (B) Spearman's correlation analysis was used to determine the correlation between the expression levels of p70S6K1 and miR-128 by SPSS software (Spearman's correlation analysis, r = −0.741; P<0.001).

To determine whether the reduced miR-128 expression correlates with the levels of p70S6K1 expression in tumor tissues, protein expression of p70S6K1 in glioma and normal brain tissues was analyzed by immunoblotting. The results revealed that the protein levels of p70S6K1 in glioma tissues were dramatically higher than those in normal brain tissues (Figure 3A). As shown in Figure 3B, Spearman's correlation test showed a significant inverse correlation of the miR-128 and p70S6K1 expression levels in glioma tissues with Spearman's correlation, r = −0.741 (P<0.001), confirming that lower expression levels of miR-128 were significantly associated with higher levels of p70S6K1 protein expression in the same set of glioma tissues.

### Overexpression of miR-128 inhibited HIF-1α and VEGF expression

**Figure 4:**
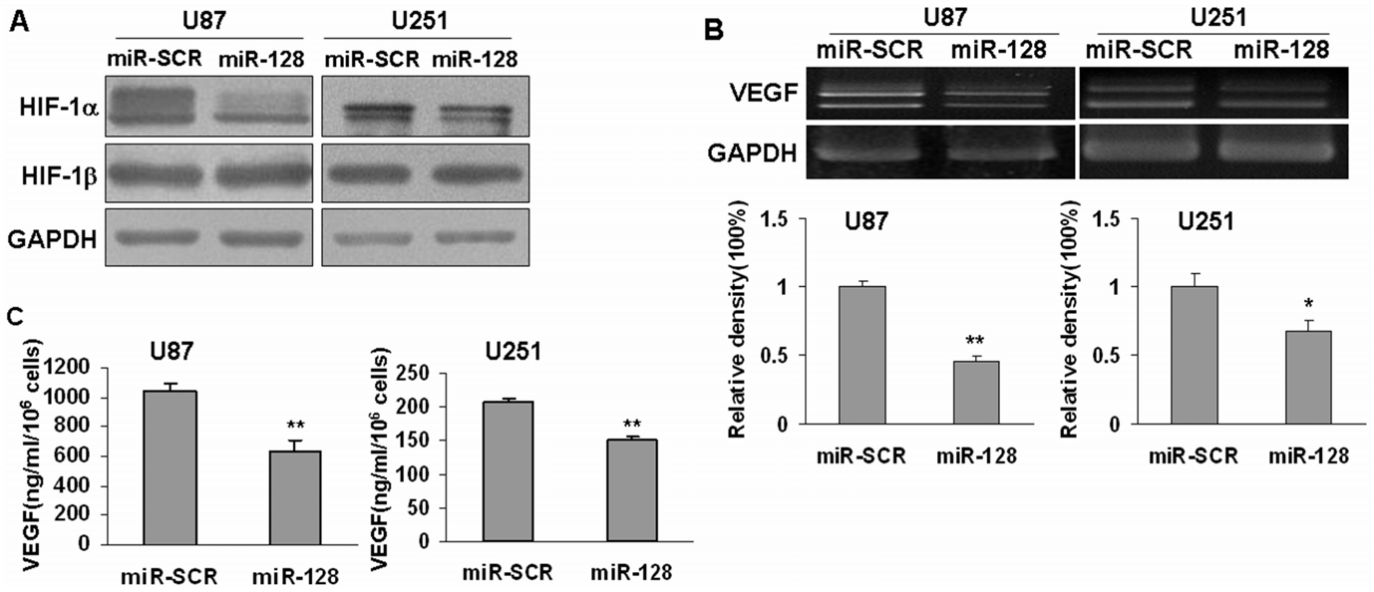
Overexpression of miR-128 inhibited HIF-1 and VEGF expression. Stable U87 and U251 cell lines expressing scrambled miRNA (SCR) or miR-128 were established. (A) Levels of HIF-1α, HIF-1β, and GAPDH protein expression were detected by immunoblotting (B) VEGF mRNA levels were detected by semi-quantitative RT-PCR assay. Asterisk and double asterisks indicate significant difference when compared to U87 cell expressing miR-SCR or U25 cell expressing miR-SCR at P<0.05 or at P<0.01, respectively. (C) VEGF protein levels were detected by ELISA assay. Double asterisks indicate significant difference when compared to the control (P<0.01).

Hypoxia-inducible factor 1 (HIF-1) is a heterodimeric transcription factor composed of HIF-1α and HIF-1β subunits [28]. HIF-1β protein is constitutively expressed in most human cells. In contrast, HIF-1α expression is induced in the cells by hypoxia, growth factors, and oncogenes [29], [30]. High levels of HIF-1α expression are observed in many human cancers, and correlated with tumor growth. Previous results showed that p70S6K1 was the upstream regulator of HIF-1α expression [15], [17], [22]. To determine whether suppression of p70S6K1 expression due to miR-128 overexpression affects HIF-1 expression in glioma, we found that HIF-1α but not HIF-1β protein levels were inhibited in U87 and U251 cells stably expressing miR-128 when compared to cells stably expressing miR-SCR (Figure 4A). VEGF, the critical regulator of angiogenesis, is mainly regulated by HIF-1α through the binding to the hypoxia-responsive element of VEGF promoter region. In consistent with decreased HIF-1α levels, the VEGF levels in miR-128-expressing U87 and U251 cells were down-regulated to 55% and 30%, respectively (Figure 4B). Moreover, VEGF protein levels in the medium were tested by ELISA as previously described [15], and the results showed that overexpression of miR-128 inhibited VEGF protein production (Figure 4C). These results suggest that forced expression of miR-128 inhibited HIF-1α and VEGF expression via p70S6K1 signaling.

### Forced expression of p70S6K1 restored the inhibitory effects of miR-128

**Figure 5:**
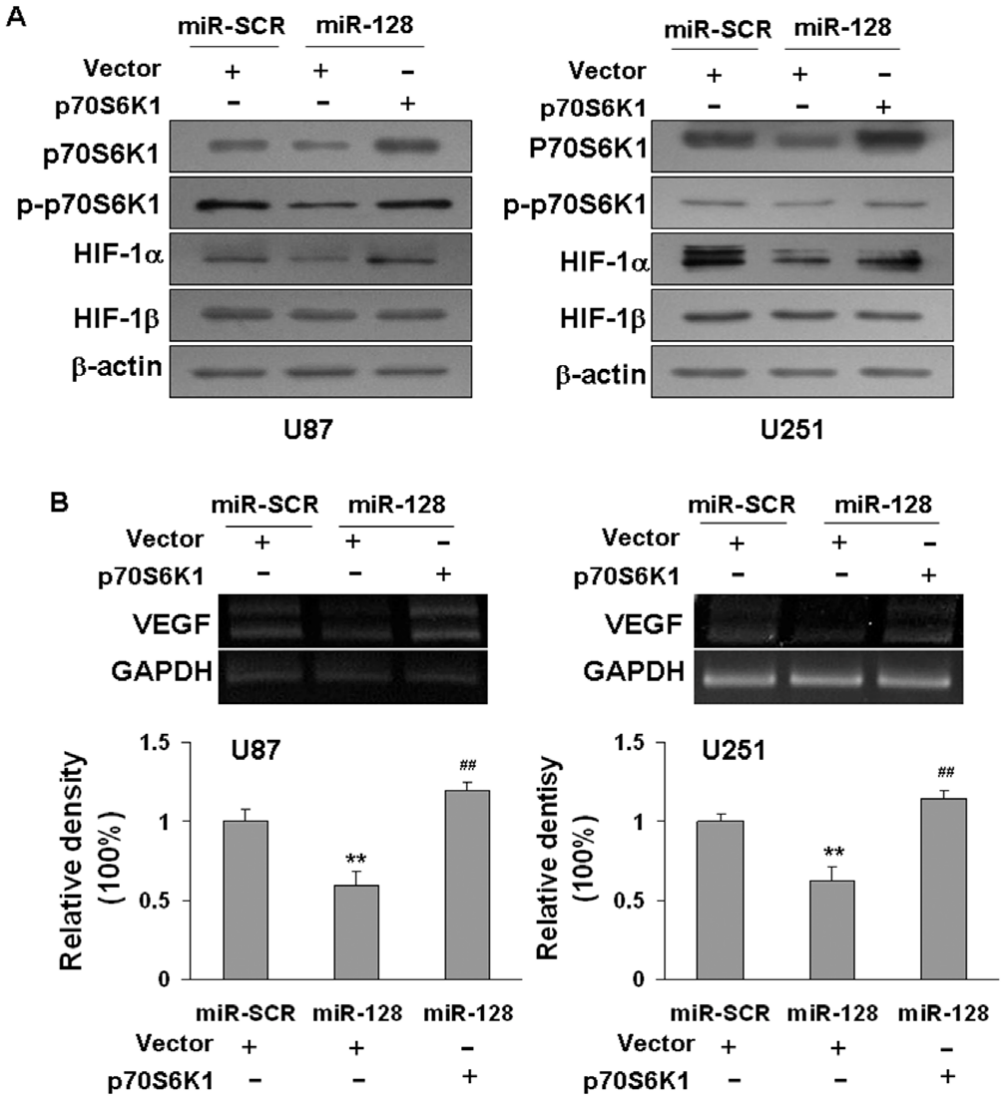
Overexpression of p70S6K1 restored the inhibitory effects of miR-128 in HIF-1 and VEGF expression. U87 and U251 cells stably expressing miR-SCR or miR-128 were transfected with p70S6K1 cDNA plasmid without 3′ UTR at 2 µg per well. After transfection for 48 h, the levels of specific proteins were analyzed by immunoblotting. (A) Overexpression of p70S6K1 increased levels of p70S6K1, phospho-p70S6K1, HIF-1α and HIF-1β protein expression. (B) Forced expression of p70S6K1 restored VEGF mRNA expression inhibited by miR-128. The levels of VEGF and GAPDH were analyzed by semi-quantitative RT-PCR and normalized to the control level. Double asterisks indicate significant difference of VEGF levels between the control and miR-128 treatment (P<0.01). Double hashes indicate significant difference of VEGF in the cells with versus without p70S6K1 overexpression (P<0.01).

As we showed above, p70S6K1 is a direct target of miR-128. Therefore, we wondered whether forced expression of p70S6K1 is enough to reverse the expression of miR-128-inhibited downstream molecules. P70S6K1 cDNA plasmid without 3′UTR region was transfected into miR-128- or miR-SCR-expressing cells. As shown in Figure 5A, the decreased level of p70S6K1 by miR-128 expression was rescued via the introduction of p70S6K1 cDNA. Similarly, ectopic expression of p70S6K1 also restored miR-128-inhibited HIF-1α and VEGF expression in both U87 and U251 cells (Figures 5A and 5B), indicating that miR-128 inhibits HIF-1α and VEGF expression by targeting p70S6K1.

### MiR-128 attenuated cell proliferation and tumor growth, and decreased p70S6K1 protein level in tumor xenografts

**Figure 6:**
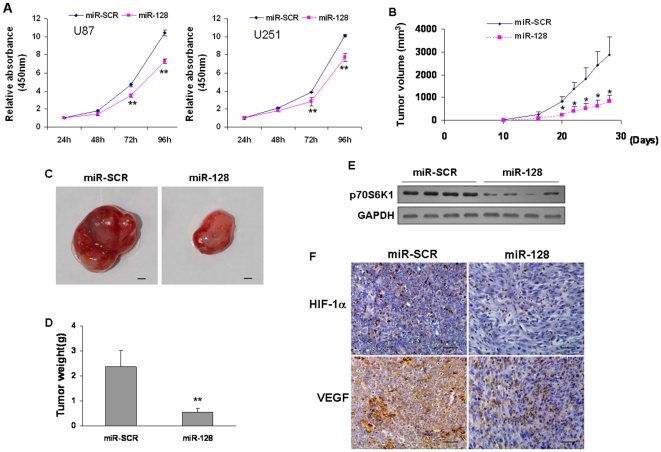
Overexpression of miR-128 inhibited cell proliferation and tumor growth. (A) U87 and U251 cells were transiently transfected with miR-SCR or pre-miR-128 precursor. After the transfection for 24 h, cells were trypsinzed and seed into 96-well plate (2000 cells/well). Cell proliferation was determined after 24, 48, 72, and 96 h by using Cell Counting Kit-8 (CCK-8) to detect the absorbance at 450 nm. Values represent means ± standard deviation (SD). Double asterisks indicate significant difference when compared to the miR-SCR group (P<0.01). (B) Nude mice were injected subcutaneously with 5×10^6^ U87 cells stably expressing miR-128 (miR-128) or miR-SCR. Each treatment group contained 8 tumors. When the xenografts were visible, the width and length of tumors were measured. Asterisk indicates significant difference when compared to the miR-SCR group (P<0.05). (C) The mice were euthanized on Day 28 and the xenografts were trimmed out. Representative tumors from each group were shown. Scale bar, 2 mm. (D) Overexpression of miR-128 significantly inhibited tumor growth *in vivo*. The tumor weight was measured for each xenograft. After 28 days, the weight of tumors from miR-128 group decreased by 75% compared to SCR group. Double asterisks indicate significant difference when compared to the miR-SCR group (P<0.01). (E) The levels of p70S6K1 from the tumor tissues of miR-128-expressing group were much higher than that of SCR control group by immunoblotting assay. (F) The expression levels of HIF-1α and VEGF were analyzed in tumor tissues by immunohistochemistry with representative images showed. Magnification, ×200.

P70S6K1 signaling pathway plays an important role in regulating cell cycle and cell proliferation [31], [32]. To study whether miR-128 can regulate cell growth by targeting p70S6K1, cell proliferation was analyzed by Cell Counting Kit-8 (CCK-8) assay. When compared to miR-SCR treatment, overexpression of miR-128 in U87 and U251 cells inhibited cell proliferation after the culture for 3–4 days, suggesting that miR-128 affects cell growth *in vitro* (Figure 6A). To further clarify the inhibitory effect of miR-128 on tumor growth *in vivo*, U87 cells stably expressing miR-128 were injected subcutaneously into both flanks of the nude mice. Cells stably expressing miR-SCR were used as negative control. The lengths and widths of tumors were measured when the xenografts were visible on Day 18, and the volumes of tumors were calculated. As shown in Figure 6B, the size of xenografts from miR-128-expressing cells was smaller than that from miR-SCR control group after 20 days of implantation. After 28 days, the xenografts were trimmed out, and tumor weight was measured. Forced expression of miR-128 in U87 cells attenuated tumor growth by 75% when compared to miR-SCR control group (Figures 6C and 6D). Furthermore, the protein levels of p70S6K1 in xenografts from miR-128-expressing cells were much lower than that from miR-SCR control cells (Figure 6E), confirming that miR-128 overexpression suppressed p70S6K1 expression *in vivo*. The expression levels of HIF-1α and VEGF in the tumor sections were also higher in miR-SCR control group than those in miR-128-expressing group (Figure 6F). These results indicate that miR-128 inhibits cell proliferation and tumor growth.

### MiR-128 inhibited angiogenesis in nude mice

**Figure 7:**
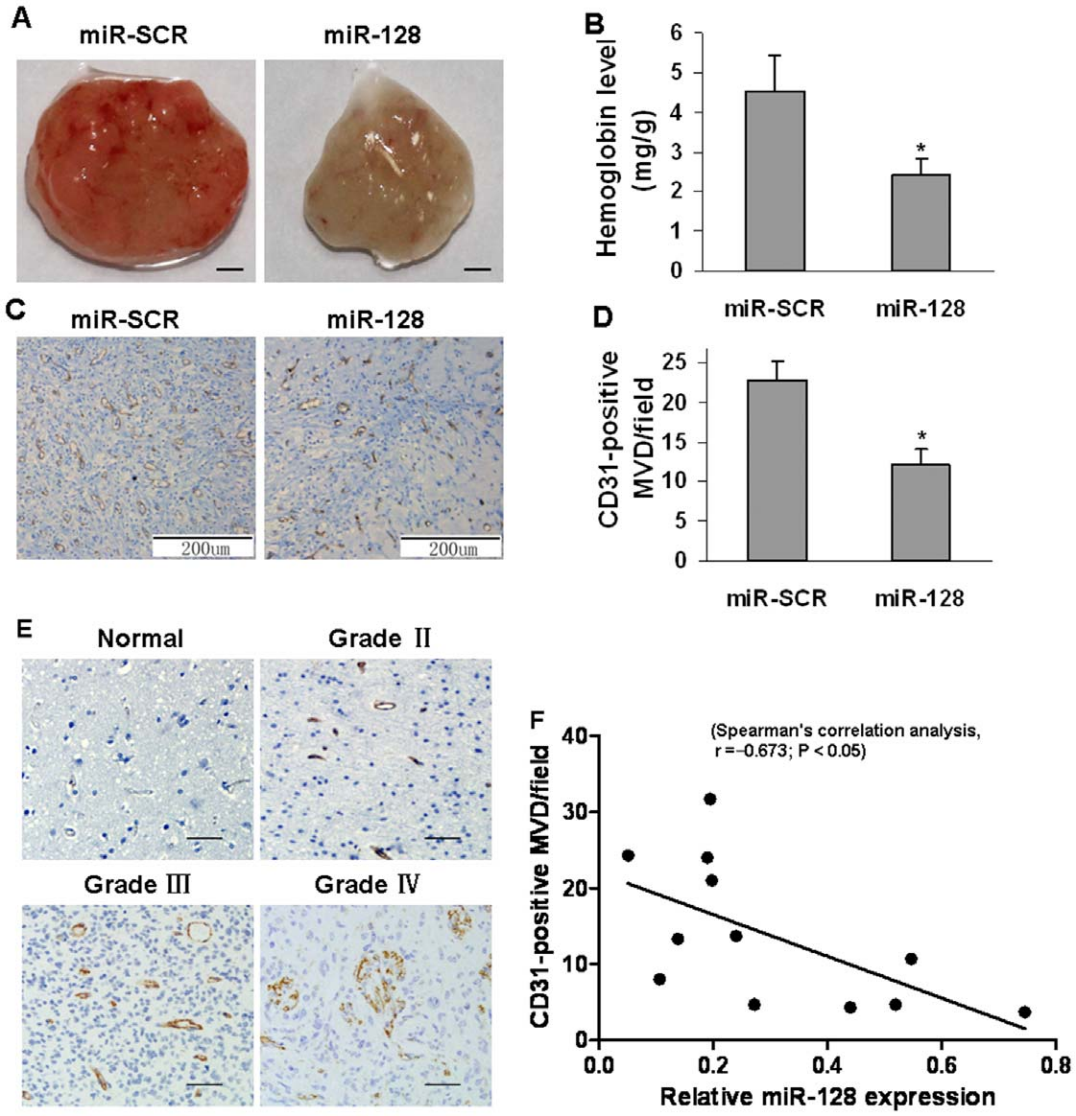
Overexpression of miR-128 suppressed tumor angiogenesis *in vivo*. U87 cells stably expressing miR-128 or miR-SCR were trypsinized and resuspended in serum-free medium at 2×10^6^cells/ml, and aliquots of the cells (0.1 ml) were mixed with 0.2 ml of growth-factor-reduced Matrigel, and injected subcutaneously into the nude mice. The mice were euthanized after the implantation for 11 days. (A) The representative Matrigel plugs are shown. Scale bar, 2 mm. (B) The hemoglobin levels in 8 tumors from miR-128 group decreased to 50% compared to SCR control group. Asterisk indicates significant difference when compared to the miR-SCR group (P<0.05). (C) Representative tumor sections stained by antibody against human CD31. Magnification, ×200. (D) CD31-positive microvessels were counted from 8 tumors in three different fields per section at ×400 magnification. Asterisk indicates significant difference when compared to the miR-SCR group (P<0.05). (E) Representative normal and different grades of glioma tumor sections stained by antibody against human CD31. Magnification, ×200. (F) Spearman's correlation test was used to analyze the correlation of miR-128 levels and CD31-positive microvessel densities from 15 glioma tumors using SPSS software.

In previous studies, we demonstrated that miR-128 down-regulated HIF-1α and VEGF expression by targeting p70S6K1. HIF-1α expression is frequently increased in many human cancers and is associated with angiogenesis induced by tumors and hypoxia [21]. Our group and others have demonstrated that p70S6K1 signaling is required for HIF-1-mediated VEGF expression in response to growth factor stimulation and oncogene activation [15]–[17]. To test whether miR-128 inhibits tumor angiogenesis, U87 cells stably expressing miR-128 or miR-SCR were mixed with Matrigel and injected into both flanks of the nude mice. The mice were sacrificed 11 days after implantation. The Matrigel plugs from the mice implanted with miR-128-expressing cells were represented in Figure 7A. The relative angiogenesis responses were assayed by measuring the hemoglobin levels. When compared to miR-SCR group, the angiogenesis responses in miR-128 group were decreased by 50% (Figure 6B). In addition, the number of CD31-positive microvessels was much lower in the sections from xenografts of miR-128-expressing U87 cells (Figures 7C and 7D), indicating that miR-128 attenuated glioma cell-inducing angiogenesis. Furthermore, we selected four cases in every grade of glioma tissues, and analyzed the number of CD31-positive microvessels in glioma specimens, the normal brain tissues were used as negative control. As shown in Figure 7E, the number of CD31-positive microvessels was much lower in normal brain tissue sections than those in glioma specimens. We analyzed CD31-positive microvessel density and miR-128 expression levels in the glioma tissues using SPSS software, and found that there is a significant inverse correlation of miR-128 levels and the CD31-positive microvessel densities in glioma tissues with Spearman's correlation, r = −0.673 (P<0.05).

## Discussion

MiRNAs function as post-transcriptional gene regulators in regulating various physiological and pathological events [33]. MiRNA abnormalities are thought to play important roles in cancer development. Recent studies have showed that many miRNAs are down-regulated in tumors when compared to normal tissues [34]. Consistent with previous studies [9], [10], we demonstrated that the expression of miR-128 in glioma tissues was down-regulated when compared to normal brain tissues. The results indicate that miR-128 is involved in pathogenesis of glioma. To determine the role and molecular mechanism of miR-128 in glioma development, we used computational bioinformatics to predict the potential targets of miR-128. Among the potential targets, we confirmed that p70S6K1 is a novel target of miR-128 by experimental method with forced expression of miR-128 suppressing p70S6K1 protein expression. In agreement with these results, overexpression of p70S6K1 rescued the inhibitory effect of miR-128 on p70S6K1 signaling pathway. Interestingly, the levels of miR-128 were negatively correlated with p70S6K1 protein levels in glioma tissues. Thus, this study is useful not only to reveal a novel mechanism of miR-128 in regulating glioma growth through targeting p70S6K1, but also to potentially use the alterations of miR-128/p70S6K1 axis for the diagnostics and treatment of glioma in the future.

Angiogenesis is the process by which new microvessels sprout from the pre-existing blood vessels. The pathophysiological processes of angiogenesis and tumor cell invasion play pivotal roles in glioma development and growth in the earliest phase and closely related to drug resistance against chemotherapy [35], [36]. Angiogenesis is required for tumor growth and metastasis. Our previous studies have showed that p70S6K1 played an important role in regulating HIF-1α and VEGF expression [15], [22]. Here we found that miR-128 overexpression can inhibit HIF-1α and VEGF expression through targeting p70S6K1. Consistent with these *in vitro* experiments, forced expression of miR-128 attenuated tumor growth and angiogenesis in nude mice. Furthermore, our results showed that miR-128 expression levels were inversely correlated with the CD31-positive microvessel densities in glioma tumor tissues. These findings indicated a new role and mechanism of miR-128 in angiogenesis and tumor growth.

In summary, we identified that miR-128 inhibited tumorigenesis and angiogenesis through targeting p70S6K1 and suppressing p70S6K1downstream molecules such as HIF-1 and VEGF. This study identified a link between miR-128 and p70S6K1 axis, which plays vital role in glioma angiogenesis, and provided a potential new target in glioma diagnostics and therapy in the future.
